# Image dataset of healthy and infected fig leaves with Ficus leaf worm

**DOI:** 10.1016/j.dib.2023.109958

**Published:** 2024-01-01

**Authors:** Saad Jabir Hafi, Mohammed Abdallazez Mohammed, Dhafar Hamed Abd, Haya Alaskar, Nawaf R. Alharbe, Sam Ansari, Salah A. Aliesawi, Abir Jaafar Hussain

**Affiliations:** a University of Anbar, Upper Euphrates Basin Developing Center, Iraq; b University of Karbala, College of Computer Science and Information Technology, Department of Computer Science, Iraq; c College of Computer Science and Information Technology, University of Anbar, Ramadi, Iraq; d Computer Science Department, Prince Sattam bin Abdulaziz University, Alkharj, Saudi Arabia; e College of Computer Science and Engineering, Taibah University, Medina, Saudi Arabia; f Department of Electrical Engineering, University of Sharjah, Sharjah, United Arab Emirates; g School of Computer Science and Mathematics, Byrom Street, Liverpool John Moores University, Liverpool, L33AF, United Kingdom

**Keywords:** Pattern recognition, Computer vision, Machine learning, Deep learning, Classification, Ficus Leaf Worm, Fig tree

## Abstract

This work presents an extensive dataset comprising images meticulously obtained from diverse geographic locations within Iraq, depicting both healthy and infected fig leaves affected by Ficus leafworm. This particular pest poses a significant threat to economic interests, as its infestations often lead to the defoliation of trees, resulting in reduced fruit production. The dataset comprises two distinct classes: infected and healthy, with the acquisition of images executed with precision during the fruiting season, employing state-of-the-art high-resolution equipment, as detailed in the specifications table. In total, the dataset encompasses a substantial 2,321 images, with 1,350 representing infected leaves and 971 depicting healthy ones. The images were acquired through a random sampling approach, ensuring a harmonious blend of balance and diversity across data emanating from distinct fig trees. The proposed dataset carries substantial potential for impact and utility, featuring essential attributes such as the binary classification of infected and healthy leaves. The presented dataset holds the potential to be a valuable resource for the pest control industry within the domains of agriculture and food production.

Specifications Table

**Table:** 

Subject	Computer Science.
Specific subject area	Computer Vision, Pattern Recognition, Machine Learning, Deep Learning.
Data format	Raw.
Type of data	Image.
Data collection	• Images of fig leaves were meticulously captured across various regions in Iraq, categorically representing two classes: infected and healthy. The dataset provides invaluable information pertaining to high-resolution leaf images, which is instrumental in the precise identification of infections. These fig leaves were photographed during the peak of their fruiting season, ensuring the utmost confidence in the accuracy of infection identification. • The dataset, encompassing two distinct classes, comprises a total of 2321 images. Within this dataset, there are 1350 images belonging to the 'infected' class, and 971 images representing the 'healthy' class. The fig leaves were captured employing a digital camera device of the following specifications: - Brand**:** Sony DSC—H300/BM. - Maximum Webcam image resolution: 20.1 MP. - Photo Sensor Size: 1/2.3-inch. - Image stabilization: optical.
Data source location	• Institution: University of Anbar and University of Karbala. • Region/State: Anbar Governorate. • Cities: Ramadi, Haditha, Rawa, Khaldia, Faloga, and Heet. • Country: Iraq • Latitude and longitude: Between these two coordinates ➢ 33° 25′ 15″ N 043° 18′ 26″ E ➢ 33° 21′ 00″ N 043° 47′ 00″ E
Data accessibility	Repository name: Mendeley Data. Data identification number: DOI: 10.17632/f7dk2yknff.2 Direct URL to data: https://data.mendeley.com/datasets/f7dk2yknff/2

## 1 Value of the Data

• This dataset holds significant potential in early infestation detection, thus preventing detrimental impacts on production.
• Early identification of infection is paramount due to the profound repercussions of Ficus leafworm infestations on both leaves and production. Additionally, it mitigates the substantial effort and costs incurred in managing these infestations, ultimately leading to a more efficient and productive yield.
• Traditional methods for identifying fig leaf infections necessitate expertise on-site, often resulting in substantial labor costs and efforts.
• The development of an intelligent classification model for precise infection detection represents a pivotal initiative, offering substantial economic advantages.
• The proposed dataset holds potential utility for the computer science community, especially in the fields of computer vision, machine learning, and deep learning. It serves as a valuable resource for developing robust classification models capable of accurately detecting infections.

## 2 Data Description

**Fig. 1:**
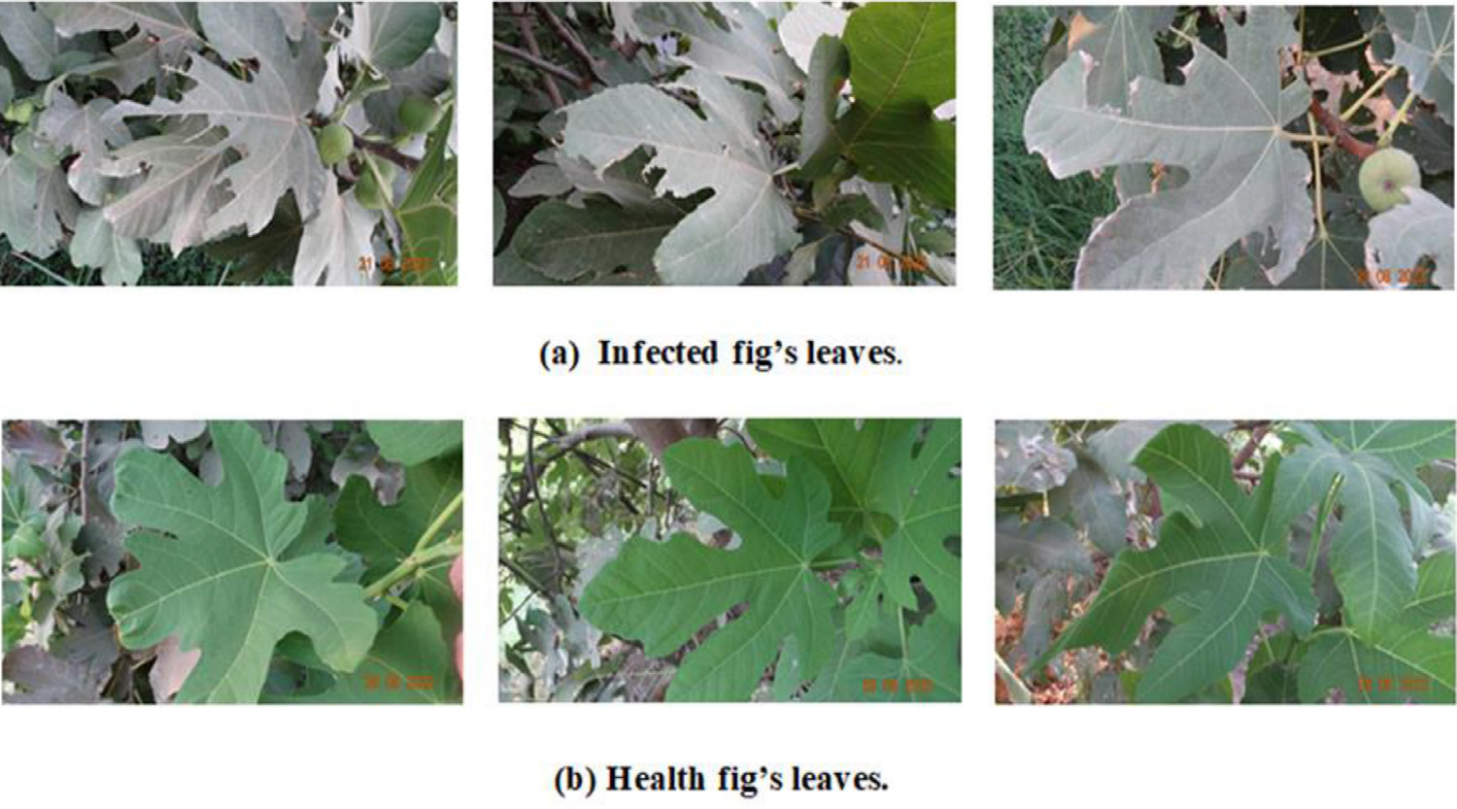
Exemplars of the encompassed classes are evidenced as follows: (a) Leaves of figs exhibiting infection, (b) Leaves of figs demonstrating robust health.

The dataset is organized into two folders, infected and healthy. The infected folder contains 1350 JPG format images, while the healthy folder contains 971 images. All the images in the dataset are of high resolution, measuring either 768 × 1365 pixels or 1365 × 768 pixels. Due to the substantial image quality, the dataset's size amounts to 681 megabytes (MB). The fig leaves have been captured as illustrated in Fig. 1.

## 3 Experimental Design, Materials and Methods

**Fig. 2:**
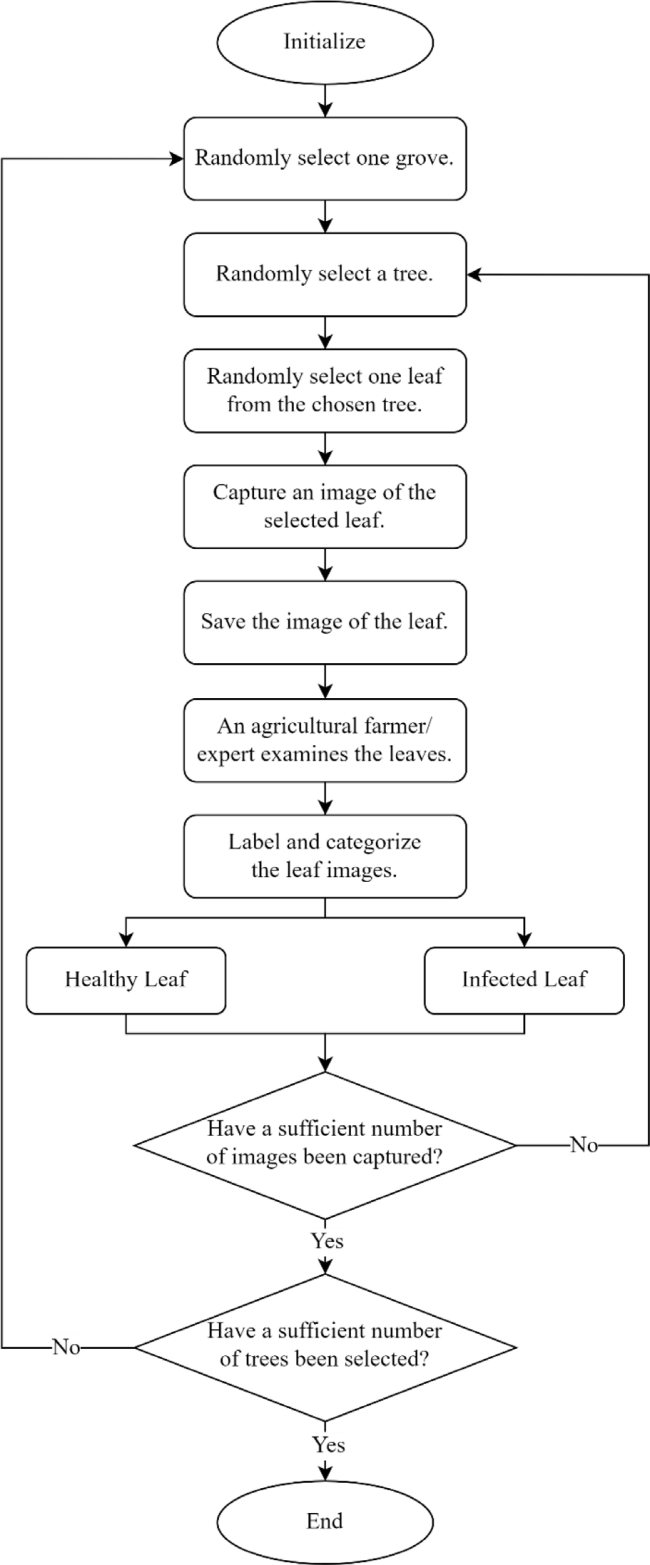
Workflow for image acquisition of leaf specimens across multiple classes via randomized grove and tree selection for each class.

The acquisition of images for both infected and healthy leaves is a time-consuming process, as indicated in [1]. The presented methodology adheres to a systematic workflow based on a random leaf selection approach, as depicted in Fig. 2. In the proposed approach, the selection of constituents from the population of infected and healthy leaves followed a uniform distribution, ensuring an equal probability of selection for each.

Data diversity was preserved through the implementation of a random leaf selection approach, wherein both the leaves chosen for photography and the trees from which they were selected were determined at random.

In the post-image acquisition process for infected leaves, the images were transferred from the camera's memory to an external hard drive and organized within a folder labeled "infected." Subsequently, the acquisition of images for healthy leaves commenced following the removal of the transferred infected images. It is worth noting that leaves with excessive dust or other forms of visual interference were not captured extensively, as they introduce unwanted noise into the dataset. All photographic activities were carried out with the knowledge and consent of the respective farm owners, ensuring the ethical and legal aspects of data collection were adhered to.

## Limitations

Not applicable.

## Ethics Statement

The authors of this dataset have diligently adhered to the ethical prerequisites for publication in Data in Brief. It can be confirmed that the current study does not involve human subjects, animal experiments, or data collected from social media platforms.

## CRediT authorship contribution statement

**Saad Jabir Hafi:** Conceptualization. **Mohammed Abdallazez Mohammed:** Data curation, Writing – original draft. **Dhafar Hamed Abd:** Investigation. **Haya Alaskar:** Writing – review & editing. **Nawaf R. Alharbe:** Writing – review & editing. **Sam Ansari:** Writing – review & editing. **Salah A. Aliesawi:** Writing – review & editing. **Abir Jaafar Hussain:** Writing – review & editing, Supervision.

## Data Availability

fig leaves dataset (Original data) (Mendeley Data).
